# Glycosylation enables aesculin to activate Nrf2

**DOI:** 10.1038/srep29956

**Published:** 2016-07-15

**Authors:** Kyun Ha Kim, Hyunsu Park, Hee Jin Park, Kyoung-Hwa Choi, Ruxana T. Sadikot, Jaeho Cha, Myungsoo Joo

**Affiliations:** 1 School of Korean Medicine, Pusan National University, Yangsan 626-870, Korea; 2 Department of Microbiology, Pusan National University, Busan 609-735, Korea; 3 Section of Pulmonary and Critical Care Medicine, Atlanta Veterans Affairs Medical Center, Emory University, Decatur, GA30033, USA

## Abstract

Since aesculin, 6,7-dihydroxycoumarin-6-*O*-β-glucopyranoside, suppresses inflammation, we asked whether its anti-inflammatory activity is associated with the activation of nuclear factor-E2-related factor 2 (Nrf2), a key anti-inflammatory factor. Our results, however, show that aesculin marginally activated Nrf2. Since glycosylation can enhance the function of a compound, we then asked whether adding a glucose makes aesculin activate Nrf2. Our results show that the glycosylated aesculin, 3-*O*-β-d-glycosyl aesculin, robustly activated Nrf2, inducing the expression of Nrf2-dependent genes, such as heme oxygenase-1, glutamate-cysteine ligase catalytic subunit, and NAD(P)H quinone oxidoreductase 1 in macrophages. Mechanistically, 3-*O*-β-d-glycosyl aesculin suppressed ubiquitination of Nrf2, retarding degradation of Nrf2. Unlike aesculin, 3-*O*-β-d-glycosyl aesculin significantly suppressed neutrophilic lung inflammation, a hallmark of acute lung injury (ALI), in mice, which was not recapitulated in Nrf2 knockout mice, suggesting that the anti-inflammatory function of the compound largely acts through Nrf2. In a mouse model of sepsis, a major cause of ALI, 3-*O*-β-d-glycosyl aesculin significantly enhanced the survival of mice, compared with aesculin. Together, these results show that glycosylation could confer the ability to activate Nrf2 on aesculin, enhancing the anti-inflammatory function of aesculin. These results suggest that glycosylation can be a way to improve or alter the function of aesculin.

Aesculin, 6,7-dihydroxycoumarin-6-*O*-β-glucopyranoside, is a coumarin glucoside found in various plants including the stem barks of *Fraxinus rhynchophylla* Dence (*Oleaceae*)^1^ that have been used for treating inflammatory diseases in Asian traditional medicine^2^. Concordantly, aesculin has been reported as an anti-oxidant and anti-inflammatory molecule^3^. In mouse disease models, aesculin reduces oxidative damage in the liver^4^ and inflammation in the lung^5^. The anti-inflammatory activity of aesculin is attributed to inhibiting NF-κB^5^. However, the precise mechanism for the anti-inflammatory activity of aesculin remains unknown, because aesculin may not suppress NF-κB in other conditons^6^.

Cumulative evidence shows that nuclear factor-E2-related factor 2 (Nrf2) is a critical factor in regulating inflammation^7^,^8^. Nrf2 was originally identified as a key transcription factor that regulates the expression of phase 2 detoxifying and antioxidant enzymes such as glutamate-cysteine ligase catalytic subunit (GCLC), NAD(P)H:quinine oxidoreductase-1 (NQO1), and heme oxygenase-1 (HO-1)^9^,^10^. In normal conditions, Nrf2 is negatively regulated by Kelch-like ECH-associated protein 1 (Keap1). Keap1 physically binds to Nrf2 in cytoplasm, mediating ubiquitination and thus rendering degradation of Nrf2^11^. In an oxidative environment, reactive oxygen species (ROS) are generated, which disrupt the inhibitory function of Keap1, resulting in Nrf2 accumulating in the nucleus, indicative of activated Nrf2^12^. The anti-inflammatory function of Nrf2 has been studied in various inflammatory lung disease mouse models including acute lung injury (ALI)^13^ and chronic obstructive pulmonary disease (COPD)^14^. Therefore, activating Nrf2 could be an effective therapeutic option to suppress inflammatory responses in diseases.

Since aesculin shows an anti-inflammatory effect, we hypothesized that this effect is associated with aesculin activating Nrf2. Given that glycosylation of baicalein enhances the anti-inflammatory activity of the compound^15^, we examined whether adding a glucose to aesculin affects Nrf2 activity. Our results show that the glycosylated aesculin was more effective than aesculin in activating Nrf2 and in protecting mice from ALI and sepsis. Biochemical and genetic experiments show that the anti-inflammatory function of the glycosylated aesculin was mainly mediated by Nrf2. Our results provide evidence that glycosylation can be an option to alter or enhance the function of natural compounds such as aesculin.

## Results

### Glycosylation of aesculin and activation of Nrf2

**Figure 1:**
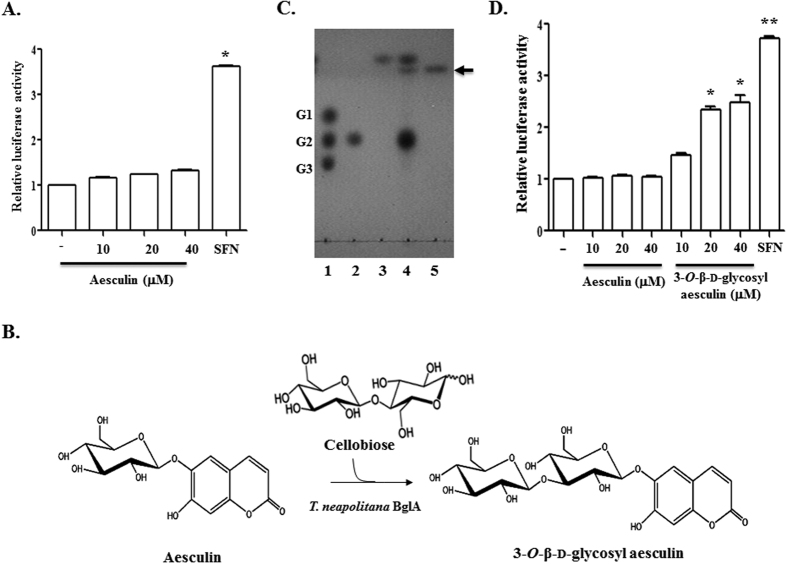
The effect of aesculin and its transglycosylated product on Nrf2 activation. Nrf2-dependent transcriptional activity was determined by the luciferase activity of Nrf2-reporter cells that were treated with aesculin alone (**A**) or along with 3-*O*-β-d-glycosyl aesculin (**D**). Assay was performed in triplicate and normalized by the amount of total protein. Representative results of three independent experiments are shown. *P and **P were <0.001, compared with untreated controls. (**B**) Schematic summarizes the transglycosylation reaction of aesculin catalyzed by the *T. neapolitana* BglA (β-glucosidase) mutant. (**C**) The transglycosylated aesculin was analyzed by TLC. Standard markers for glucose (G1), cellobiose (G2), and cellotriose (G3) are shown in lane 1. Cellobiose (lane 2) and aesculin (lane 3) were mixed in the presence of the modified BglA for aesculin transglycosylation (lane 4). The resultant product, 3-*O*-β-d-glycosyl aesculin, was purified, which is indicated by arrow (lane 5).

To investigate if the anti-inflammatory activity of aesculin is mediated through Nrf2, we treated with various amounts of aesculin Nrf2 reporter cells, which harbor a 1.0 kb-long of NQO-1 promoter fused with a fire fly luciferase gene in RAW 264.7 cells^16^. At 16 h after treatment, cells were harvested for measuring Nrf2-driven luciferase activity. As shown in Fig. 1A, while sulforaphane, a potent activator of Nrf2^17^, increased luciferase activity, aesculin did not, suggesting that aesculin does not activate Nrf2 and that the anti-inflammatory activity of aesculin may not involve Nrf2.

Since glycosylation can enhance the function of baicalein^15^, we tested the possibility that glycosylation increases the ability of aesculin to activate Nrf2. First, aesculin was transglycosylated, as illustrated in Fig. 1B. TLC analysis of reactants identified a major product, whose yield was approximately 15% according to densitometric analysis (Fig. 1C). MALDI-TOF/MS analysis of the transglycosylated product showed a molecular-related ion peak at *m*/*z* 543 [M + Na]^+^, indicating that the product consisted of one glucose unit and aesculin. ^1^H and ^13^C NMR analysis showed that the glucosidic linkage of aesculin had typical chemical shifts for an aesculetin skeleton plus a set of signals corresponding to a glucose unit at C-6, aesculetin 6-β-d-glucopyranoside. The position for the terminal glucose linked to aesculin was determined through the glycosylation shift of the C-3′ signal of the glucose moiety in aesculin. The transglycosylated aesculin showed 21 carbon signals including those of a terminal glucose molecule (Table 1). The chemical shift of C-3′ in the glucose unit of aesculin was changed from 77.2 to 84.0 ppm, confirming that transferred glucosyl group was connected to C-3′ in the glucose unit of aesculin. In addition, ^1^H analysis revealed that the glucosyl residue was transferred to C-3′ in the glucose unit of aesculin to give the β-anomeric configuration based on the coupling constant (*J* = 7.6 Hz) of the glucose anomeric proton signal observed at 5.06 ppm. Two-dimensional HMBC spectra of the glycosylated aesculin confirmed the molecular structure of the compound (data not shown), which was identified as 3-*O*-β-d-glycosyl aesculin (or aesculin-3-*O*-β-d-glucopyranoside) (Fig. 1B). Next, we tested whether the glycosylated aesculin, 3-*O*-β-d-glycosyl aesculin, activates Nrf2. Similar to Fig. 1A, the Nrf2 reporter cells were treated with aesculin or 3-*O*-β-d-glycosyl aesculin for 16 h, and luciferase activity was measured. As shown in Fig. 1D, unlike aesculin, 3-*O*-β-d-glycosyl aesculin induced Nrf2-driven luciferase activity.

**Figure 2:**
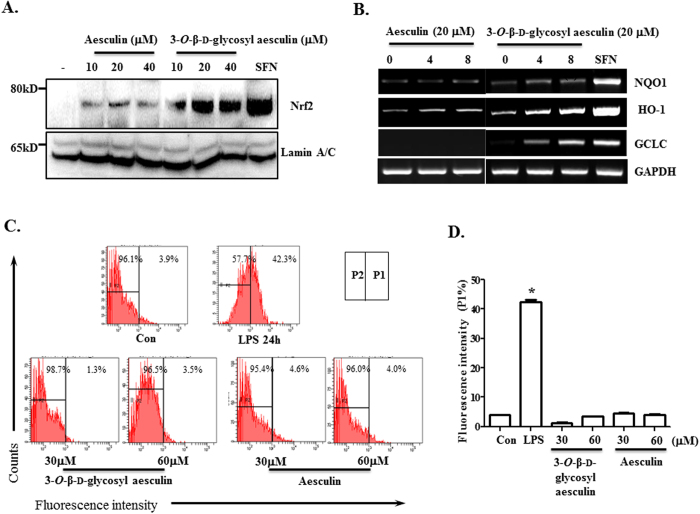
3-*O*-β-d-glycosyl aesculin is effective in activating Nrf2. (**A**) Western blot analysis was performed to reveal nuclear Nrf2 in RAW 264.7 cells treated with indicated amounts of aesculin or 3-*O*-β-d-glycosyl aesculin. The membrane was stripped and reprobed for the nuclear protein, lamin A/C. (**B**) RAW 264.7 cells were treated similar to (**A**), and the mRNA expression of Nrf2-dependent genes was analyzed by semi-quantitative RT-PCR. (**C**) RAW 264.7 cells were treated with indicated amounts of aesculin or 3-*O*-β-d-glycosyl aesculin for 16 h, and intracellular ROS of the cells were analyzed by FACS. The percentage of cells producing intracellular ROS is shown on the right panel (P1). The mean ± SEM of three independent measurements of intracellular ROS was shown in (D). *P was <0.005, compared with the untreated control.

To confirm that 3-*O*-β-d-glycosyl aesculin activates Nrf2, we performed western blot analysis of cells that were treated with aesculin or 3-*O*-β-d-glycosyl aesculin. Nuclear proteins were fractionated from the treated cells, which were analyzed by western blotting for nuclear Nrf2, indicative of activated Nrf2. As shown in Fig. 2A 3-*O*-β-d-glycosyl aesculin induced a robust accumulation of Nrf2 in the nucleoplasm, as opposed to aesculin. Consistent with these results, 3-*O*-β-d-glycosyl aesculin induced the expression of Nrf2-dependent genes (Fig. 2B). To exclude the possibility that 3-*O*-β-d-glycosyl aesculin inadvertently induces ROS, resulting in Nrf2 activation, we measured intracellular ROS by FACS analysis. As shown in Fig. 2C and Supplementary Fig. S1, while LPS strongly induced ROS, neither 3-*O*-β-d-glycosyl aesculin nor aesculin significantly generated ROS. Together, these results suggest that 3-*O*-β-d-glycosyl aesculin activates Nrf2 without ROS.

### 3-*O*-β-d-glycosyl aesculin suppresses ubiquitination of Nrf2

**Figure 3:**
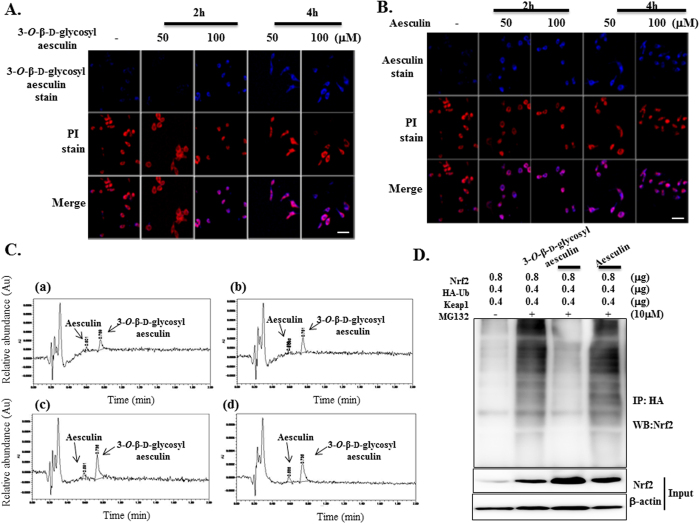
Absorption of 3-*O*-β-d-glycosyl aesculin and aesculin by RAW264.7 cells. RAW 264.7 cells were treated with indicated amounts of 3-*O*-β-d-glycosyl aesculin (**A**) or aesculin (**B**), which were then fixed and stained with PI. The compounds and cells were shown blue and red, respectively, under a confocal microscope. Cells that absorbed 3-*O*-β-d-glycosyl aesculin or aesculin appeared pink (merge). Shown are representatives of 5 randomly picked microscopic fields for each treatment (bar = 500 μm). (**C**) HPLC chromatograms of the lysate of RAW 264.7 cells treated with 50 μM (a,b) or 100 μM (c,d) of 3-*O*-β-d-glycosyl aesculin (>99% purity). Arrows indicate the peaks for 3-*O*-β-d-glycosyl aesculin (83–87%) and aesculin (13–17%). (**D**) HEK 293 cells, transfected with indicated amounts of plasmids encoding Nrf2, HA-Ub, and Keap1, were treated with 20 μM of aesculin or 3-*O*-β-d-glycosyl aesculin in the presence of MG132. HA-Ub in cytosol was precipitated by the α-HA antibody, and the immune complex was analyzed by western blotting for Nrf2.

To understand the role of the glycosylation of aesculin in Nrf2 activation, we first tested whether glycosylation affects cellular uptake. RAW 264.7 cells were treated with two different amounts of 3-*O*-β-d-glycosyl aesculin (Fig. 3A) and aesculin (Fig. 3B), which were traced up to 4 h under a confocal microscope. Cells that take up aesculin or 3-*O*-β-d-glycosyl aesculin emit blue fluorescence. In order to discern cells under the microscope, cells were stained with a nucleic acid dye PI that emits red fluorescence. Therefore, PI-stained cells that took up the chemicals appeared pink due to wave interference. As shown in Fig. 3A3-*O*-β-d-glycosyl aesculin was detectable in the cytoplasm within 2 h when administered with 100 μM or within 4 h when treated with 50 μM. However, in parallel experiments, aesculin was detectable in the cytoplasm within 2 h when administered with 50 μM (Fig. 3B), suggesting that 3-*O*-β-d-glycosyl aesculin does not have an advantage over aesculin in cellular uptake.

Next, we examined whether 3-*O*-β-d-glycosyl aesculin is stably maintained within cells by HPLC (Fig. 3C). RAW 264.7 cells were treated with 50 μM of 3-*O*-β-d-glycosyl aesculin (>99% purity) for 2 h, and the cytosol of the treated cells was prepared for HPLC analysis. HPLC analysis identified one major peak of 3-*O*-β-d-glycosyl aesculin (0.77 min) and a minor peak of aesculin (0.59 min) (a). The major 3-*O*-β-d-glycosyl aesculin peak was also detected in the cytosol of the cells treated for 4 h (b). In similar experiments with 100 μM of 3-*O*-β-d-glycosyl aesculin, a major 3-*O*-β-d-glycosyl aesculin peak and a minor aesculin peak were identified at 2 h (c) or 4 h (d) after the 3-*O*-β-d-glycosyl aesculin treatment. Based on the calculation of peak area, 3-*O*-β-d-glycosyl aesculin and aesculin constituted 83–87% and 13–17%, respectively, of total peaks. These results show that 3-*O*-β-d-glycosyl aesculin was stable within cells, suggesting the possibility that the intact structure of 3-*O*-β-d-glycosyl aesculin is associated with Nrf2 activation induced by 3-*O*-β-d-glycosyl aesculin.

Finally, we tested whether glycosylation enables aesculin to suppress the ubiquitination of Nrf2, resulting in Nrf2 activation. HEK 293 cells were transfected with plasmids encoding Nrf2, Keap1, and HA-ubiquitin (Ub) in the presence or absence of MG132, a proteasome inhibitor that blocks ubiquitin-mediated protein degradation. After treatment with 20 μM of aesculin or 3-*O*-β-d-glycosyl aesculin, the cytoplasm of the treated cells was fractionated, in which HA-Ub was precipitated with the α-HA antibody. The resultant immune complex was analyzed by western blotting for Nrf2 to reveal ubiquinated Nrf2. As shown in Fig. 3D, Keap1 mediated the ubiquitination of Nrf2 (2^nd^ lane). 3-*O*-β-d-glycosyl aesculin suppressed the ubiquitination of Nrf2 (3^rd^ lane), while aesculin failed to do so (4^th^ lane). To verify this observation, we performed similar experiments, in which Nrf2 was precipitated by α-Nrf2 antibody and analyzed by western blotting for HA-Ub to reveal the ubiquitinated Nrf2, and we obtained similar results (Supplementary Fig. S2). Together, these results suggest that 3-*O*-β-d-glycosyl aesculin activates Nrf2 by suppressing the ubiquitination of Nrf2.

### 3-*O*-β-d-glycosyl aesculin ameliorates neutrophilic lung inflammation in an Nrf2 dependent manner

**Figure 4:**
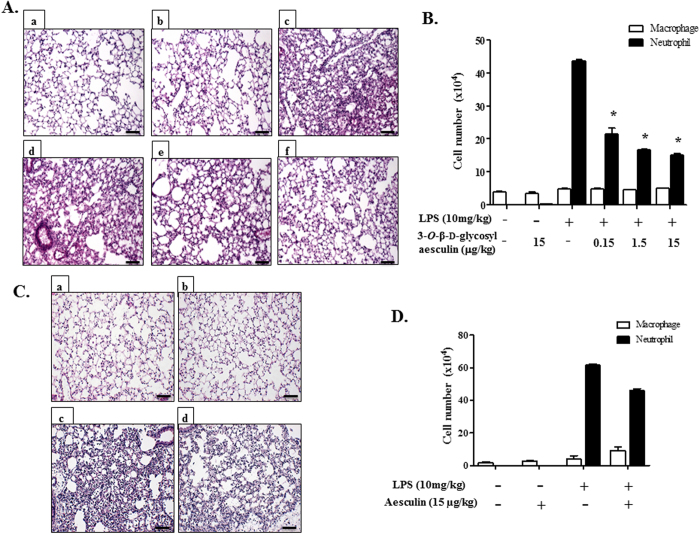
3-*O*-β-d-glycosyl aesculin suppresses neutrophilic lung inflammation in ALI mice. (**A**) C57BL/6 mice (n = 5 per group) received an i.p. PBS (a,b) or an i.p. LPS (10 mg/kg body weight; (c–f) and 2 h later 0.15 μg/kg (d), 1.5 μg/kg (e), or 15 μg/kg (f) body weight of an i.t. 3-*O*-β-d-glycosyl aesculin. Lung sections were HE stained for histological examination (magnification ×100). Shown are representatives of at least five different areas of a lung (bar = 100 μm). (**B**) BAL was performed for counting neutrophil and macrophages in the lungs of the mice treated as in (**A**). Data represent the mean ± SEM of three independent counting. *P was <0.05, compared with LPS-treated mice. (**C**) In parallel experiments, mice (n = 5 per group) received an i.p. PBS (a,b) or an i.p. LPS (c,d) and 2 h later 15 μg/kg of an i.t. aesculin (b,d). Lung sections were analyzed similar to (**A**). (**D**) BAL was performed for counting neutrophil and macrophages in the lungs of the mice treated as in (**C**). Data represent the mean ± SEM of three independent counting. There was no significant reduction in neutrophil infiltration, compared to LPS-treated mice.

Since 3-*O*-β-d-glycosyl aesculin activated Nrf2, we examined whether 3-*O*-β-d-glycosyl aesculin suppresses lung inflammation via Nrf2. First, we tested whether 3-*O*-β-d-glycosyl aesculin suppresses neutrophilic lung inflammation in an LPS-induced acute lung injury mouse model (Fig. 4A). C57BL/6 mice (n = 5 per group) received an i.p. PBS (a and b) or an i.p. LPS (10 mg/kg body weight; c, d, e, and f) and 2 h later 0.15, 1.5, and 15 μg/kg body weight of i.t. 3-*O*-β-d-glycosyl aesculin (d, e, and f, respectively). Histologic analyses of the lung show that LPS induced cellular infiltration and hyaline changes in the lung (c), hallmarks of ALI^18^, which were suppressed by 3-*O*-β-d-glycosyl aesculin (d, e, and f). Bronchoalveolar lavage (BAL) was performed for measuring total cells (Supplementary Fig. S3A) and macrophages and neutrophils infiltrated to the lung (Fig. 4B). The results show that while 0.15 μg/kg of 3-*O*-β-d-glycosyl aesculin was capable of suppressing neutrophil infiltration, 1.5 μg/kg of 3-*O*-β-d-glycosyl aesculin was as effective as 15 μg/kg in suppressing neutrophil infiltration. Since 15 μg/kg of 3-*O*-β-d-glycosyl aesculin was the highest dose in this experiment that suppressed neutrophilic lung inflammation, we tested whether the same amount of aesculin (15 μg/kg) is able to exert this anti-inflammatory effect in the mouse lungs. As shown in Fig. 4C, however, aesculin did not ameliorate lung inflammation (c and d). Consistent with this result, total cells (Supplementary Fig. S3B) and macrophages and neutrophils (Fig. 4D) infiltrated to the lung were not significantly decreased by aesculin either. These results suggest that 3-*O*-β-d-glycosyl aesculin, but not aesculin, suppresses neutrophilic lung inflammation.

**Figure 5:**
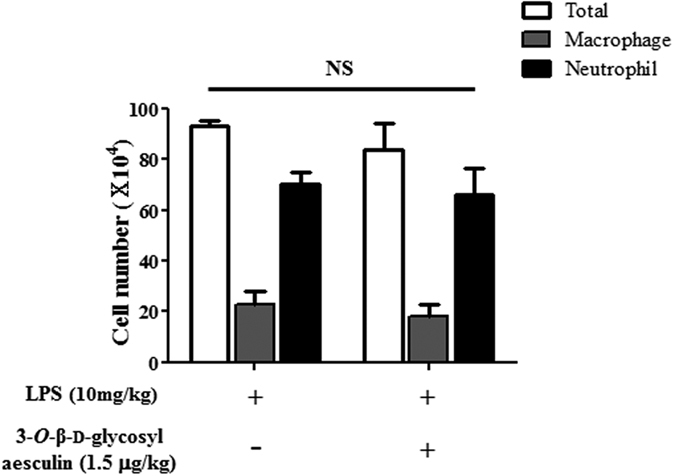
Anti-inflammatory activity of 3-*O*-β-d-glycosyl aesculin is dependent on Nrf2. Nrf2 KO mice (n = 6 per group) received an i.p. LPS (10 mg/kg body weight), with or without 3-*O*-β-d-glycosyl aesculin (1.5 μg/kg body weight). The numbers of total cells, neutrophils, and macrophages in BAL fluid were counted, which are shown in empty, gray, and dark columns, respectively. No statistical significance (NS) between 3-*O*-β-d-glycosyl aesculin-treated and untreated control groups was found.

Next, we determined whether the anti-inflammatory function of 3-*O*-β-d-glycosyl aesculin is dependent on Nrf2. Since 1.5 μg/kg of 3-*O*-β-d-glycosyl aesculin was as effective as 15 μg/kg, we administered an i.p LPS (10 mg/kg) and 2 h later 1.5 μg/kg of i.t. 3-*O*-β-d-glycosyl aesculin to Nrf2 KO mice (n = 6 per group). BAL was performed, and inflammatory cells in the BAL fluid were scored 24 h after LPS treatment. As shown in Fig. 5, LPS induced neutrophilic inflammation in the lungs, which was, however, not significantly suppressed by 3-*O*-β-d-glycosyl aesculin. Together, these results suggest that 3-*O*-β-d-glycosyl aesculin suppresses neutrophilic lung inflammation, which is mostly dependent on Nrf2.

### 3-*O*-β-d-glycosyl aesculin post-treatment improves the survival of mice from sepsis

**Figure 6:**
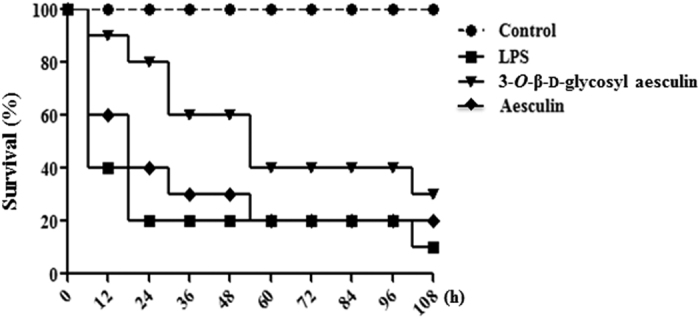
3-*O*-β-d-glycosyl aesculin treatment increases the survival of septic mice. Mice (n = 20 per group) received LPS alone (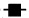) or LPS with 3-*O*-β-d-glycosyl aesculin (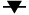) or aesculin (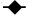), and were monitored for 4.5 days. The mortality of mice was shown as Kaplan–Meier survival curves (log-rank test, *P < 0.05).

Given that sepsis is the major cause of ALI^19^,^20^, we tested whether 3-*O*-β-d-glycosyl aesculin protects mice from sepsis in a sepsis mouse model. Septic shock was induced by i.p. injection of mice (n = 20 per group) with a lethal dose of LPS (30 mg/kg body weight) along with d-(+)-galactosamine hydrochloride (500 mg/kg body weight)^21^. At 2 h after injection, mice received an i.t. 3-*O*-β-d-glycosyl aesculin or aesculin (1.5 μg/kg), and were monitored every 12 h for 4.5 days. As shown in Fig. 6, the mortality of septic mice was about 80% within 24 h after an i.p. LPS/ d-(+)-galactosamine hydrochloride injection, and this remained so up to 4 days after injection (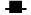). Septic mice that received aesculin showed 60% mortality within 24 h after injection, which was increased to 80% over approximately 2 days (p < 0.1, compared to LPS-treated mice) (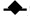). On the other hand, the mortality of septic mice that received 3-*O*-β-d-glycosyl aesculin was 20% within 24 h after injection and remained 60% for up to 4 days (p < 0.05, compared to LPS-treated mice) (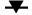). No mortality was observed in mice treated with PBS (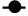). Collectively, our results suggest that 3-*O*-β-d-glycosyl aesculin protects mice from septic lung inflammation more effectively than aesculin.

## Discussion

Aesculin has been suggested as an anti-inflammatory molecule. However, the mechanism by which aesculin exerts its anti-inflammatory activity remains controversial. Therefore, we performed this study to examine our hypothesis that aesculin might exert its anti-inflammatory function via activation of Nrf2 because Nrf2 is a key factor that suppresses inflammation. Unlike our hypothesis, however, we found that aesculin was not effective in activating Nrf2. Since glycosylation can alter or enhance the function of a compound, we sought to ask whether adding a glucose to C-3′ in the glucose unit of aesculin alters the effectiveness of aesculin in activating Nrf2. We found that the glycosylated aesculin, 3-*O*-β-d-glycosyl aesculin, robustly activated Nrf2 and induced the expression of Nrf2-dependent genes. Activation of Nrf2 by 3-*O*-β-d-glycosyl aesculin was associated with suppressed ubiquitination of Nrf2. Compared to aesculin, 3-*O*-β-d-glycosyl aesculin significantly suppressed neutrophilic lung inflammation in an ALI mice mouse model, which was Nrf2 dependent. Furthermore, 3-*O*-β-d-glycosyl aesculin significantly reduced the mortality caused by sepsis in a sepsis mouse model. Therefore, our results suggest that adding a glucose can be a way to improve or alter the function of aesculin.

Our results show that aesculin gained the ability to activate Nrf2 after glycosylation. However, it is unknown how glycosylation bestowed the function on aesculin. It is conceivable that glycosylation can alter the properties of aesculin. For example, glycosylation could contribute to the solubility of aesculin, as evidenced by the reports that the glycosylation of ampelopsin^22^ and puerarin^23^ increases their solubility by 14–200 folds. Glycosylation also could affect the stability of aesculin because glycosylation of the *p*-hydroxy group of resveratrol abolishes the enzymatic oxidation induced by mushroom tyrosinase, protecting resveratrol from enzymatic oxidation^24^. However, glycosylation did not significantly affect the solubility or stability of aesculin. Aesculin is highly soluble in aqueous solutions, the solubility of which was comparable to 3-*O*-β-d-glycosyl aesculin (data not shown). In addition, the half-life, t_1/2_ (h), of 3-*O*-β-d-glycosyl aesculin was similar to aesculin because both aesculin and 3-*O*-β-d-glycosyl aesculin were stable in complete DMEM culture media at least for 72 h (data not shown). As shown in this study, 3-*O*-β-d-glycosyl aesculin and aesculin were stable in the cytosol of cells for at least 4 h. These results suggest that glycosylation does not give a benefit to aesculin in terms of solubility or stability.

Our results show that glycosylation provided an unexpected functional advantage to aesculin, the activation of Nrf2. Blocking Keap1 is necessary to activate Nrf2^10^. Inflammatory mediators or ROS generated in inflammatory milieus inactivate Keap1. Consequently, Keap1 no longer mediates the ubiquitination of Nrf2, resulting in the activation of Nrf2 and the expression of Nrf2 dependent genes^9^,^10^. Our results show that 3-*O*-β-d-glycosyl aesculin activated Nrf2 without generating ROS. Unlike aesculin, 3-*O*-β-d-glycosyl aesculin suppressed the ubiquitination of Nrf2, elicited the accumulation of Nrf2 in the nucleus, and induced the expression of mRNA of signature Nrf2-dependent genes, such as NQO-1, GCLC, and HO-1. The precise mechanism by which 3-*O*-β-d-glycosyl aesculin suppresses the ubiquitination of Nrf2 remains to be defined. As Keap1 plays a critical role in ubiquitinating Nrf2^25^, it is possible that 3-*O*-β-d-glycosyl aesculin interferes with the function of Keap1. It is conceivable that 3-*O*-β-d-glycosyl aesculin could form an adduct with Keap1, inactivating Keap1, although it is unclear whether glucosyl residue, which is chemically stable, is engaged in bonding to Keap1. Alternatively, 3-*O*-β-d-glycosyl aesculin could compete with Keap1 over Nrf2. It would be interesting to examine whether the structure of 3-*O*-β-d-glycosyl aesculin is compatible with that of Nrf2, functioning as a competitive inhibitor of Keap1. It is of note that Nrf2 is closely associated with the suppression of various inflammatory lung diseases, including ALI^13^, chronic obstructive pulmonary disease^26^, and asthma^27^ in mouse models. Consistent with the role of Nrf2 in inflammatory lung diseases, 3-*O*-β-d-glycosyl aesculin decreased neutrophilic lung inflammation, a hallmark of ALI^28^, in an LPS-induced mouse model. Although we cannot exclude the possibility that 3-*O*-β-d-glycosyl aesculin exerts its anti-inflammatory function through multiple pathways, it is highly likely that the anti-inflammatory function of the glycosylated aesculin is dependent on Nrf2 because the anti-inflammatory effect of 3-*O*-β-d-glycosyl aesculin was not found in Nrf2 KO mice.

In pharmaceutics, one of the important features for an effective drug is a high efficacy in a low-dose administration. Our results suggest that the glycosylation of aesculin can provide such a feature. Our data show that a dosage as low as 0.15 μg/kg of 3-*O*-β-d-glycosyl aesculin was effective in suppressing neutrophilic lung inflammation. A single administration of 1.5 μg/kg of the glycosylated aesculin was as effective as 15 μg/kg in suppressing lung inflammation, as assessed by lung histology and differential counting of cells infiltrated to the lung. In addition, a single administration of 1.5 μg/kg of the glycosylated aesculin significantly protected mice from succumbing to sepsis, the major cause of ALI (20% mortality of 3-*O*-β-d-glycosyl aesculin-treated mice versus 80% mortality of septic mice). Together, our results suggest that glycosylation enhances the efficacy of aesculin in suppressing inflammation, which is associated with Nrf2 activated by the glycosylated aesculin.

In this study, we examined whether aesculin executes its anti-inflammatory function by activating Nrf2, a key transcription factor that protects from various inflammatory diseases. We found that when glycosylated, aesculin activated Nrf2, effectively suppressed lung inflammation, and protected from sepsis. Experiments with Nrf2 KO mice suggest that the suppressive effect of the glycosylated aesculin on neutrophilic lung inflammation is mainly mediated by Nrf2. Although medicinal herbs can be a resource for safe drugs, the efficacy of compounds isolated from the herbs has been reported to be low. Our results provide evidence that adding a glucose can be a simple and effective way to alter or enhance the function of natural compounds.

## Materials and Methods

### Chemicals and reagents

Aesculin, cellobiose, sulforaphane, 3-(4,5-dimethylthiazol-2-yl)-2,5-diphenyltetrazolium-bromide (MTT), lipopolysaccharide (LPS; *Escherichia coli* O55:B5), and α-HA antibody (H3663) were obtained from Sigma-Aldrich (St. Louis, MO, USA). Water and methanol in high-performance liquid chromatography (HPLC)-grade were purchased from Burdick & Jackson (USA). Antibodies against Nrf2 (H-300), β-actin (C-4), and lamin A/C (H-110) were from Santa Cruz Biotechnology (Santa Cruz, CA, USA). Modified β-glucosidase (N291T) from *T. neapolitana* (BglA) was purified using Ni-NTA affinity chromatography and heat treatment^29^.

### Transglycosylation reaction and purification of glycosylated aesculin

Glycosylation of aesculin occurred in 100 mM sodium phosphate buffer (pH 7.0) with 1% (w/v) cellobiose, 2% (w/v) aesculin, and 1.8 μg of BglA (N291T) at 80 °C for 1 h. Approximately 3 ml of the reaction mixture was loaded onto a JAIGEL-W251 column (20 × 50 cm) of the recycling preparative HPLC equipped with refractive index detector (JAI, Korea) and eluted with deionized water at a flow rate of 3.0 ml/min with 30 kgf/cm^2^ of column pressure. The purified product, 3-*O*-β-d-glycosyl aesculin, was lyophilized for further analyses.

### Matrix-assisted laser desorption/ionization-time of flight (MALDI-TOF) mass spectrometry

Purified 3-*O*-β-d-glycosyl aesculin in water was mixed 1:1 (v/v) with 2,5-dihydroxybenzoic acid (1 mg/ml). The mixed solution (1 μl) was spotted onto a stainless steel plate and dried at room temperature. The mass spectra were obtained in the positive linear mode with delayed extraction (average of 150 laser shots) with a 65-kV acceleration voltage by using a Voyager DE-STR MALDI-TOF mass spectrometer (Applied Biosystems, Foster City, CA).

### Nuclear magnetic resonance analysis (NMR)

Approximately 3 mg of compound was dissolved in 250 μl of deuterium oxide (D_2_O) and dispensed into 3 mm NMR tubes. NMR spectra were obtained on a Unity Inova 500 spectrometer (Varian Inc., Palo Alto, CA, USA) that operated at 500 MHz for ^1^H and 125 MHz for ^13^C at 25 °C.

### Thin-layer chromatography (TLC)

3-*O*-β-d-glycosyl aesculin was dissolved in *n*-butanol-ethanol-water (5:3:2, v/v/v) and analyzed using TLC, Whatmann K6F silica gels (Whatmann, Maidstone, UK). After irrigating twice, the TLC plate was dried and visualized by dipping it into a solution containing 0.3% (w/v) *N*-(1-naphthyl)-ethylenediamine and 5% (v/v) H_2_SO_4_ in methanol followed by heating for 5 min at 120 °C.

### High-performance liquid chromatography (HPLC)

Aesculin and 3-*O*-β-d-glycosyl aesculin in cell lysates were analyzed by UPLC. The Acquity UPLC H Class system (Waters, Ireland) comprised a Model bioSample Manager-FTN, a Model bioQuaternary Solvent Manager, and a Model PDA eλ Detector set at 340 nm. Samples were injected with Model 701 syringes (Hamilton, Bonaduz, Switzerland), separated at 40 °C using a 1.7 μm Acquity UPLC BEH C18 column (2.1 mm × 50 mm), and eluted isocratically at a flow rate of 0.6 ml/min, with 0.5% acetic acid-methanol (85:15). The identities of aesculin and 3-*O*-β-d-glycosyl aesculin peak were assigned by co-chromatography with the authentic standard. Quantification was carried out by integration of the peak areas using the external standardization method.

### Cellular uptake of aesculin and 3-*O*-β-d-glycosyl aesculin

Cellular uptake of aesculin and 3-*O*-β-d-glycosyl aesculin was monitored under the microscope. RAW 264.7 cells (1 × 10^5^ cells/ml), a murine macrophage cell line, cultured on a glass coverslip were treated with 50 or 100 μM chemicals for 2 to 4 h. After washing with PBS, cells were fixed with 4% paraformaldehyde for 10 min, stained with propidium iodide (PI; 100 ng/ml) for 5 min, mounted with Vecta Shield (Vector Laboratories, Burlingame, CA), and examined with a Zeiss LSM 700 inverted confocal microscope (Zeiss, Oberkochen, Germany). Aesculin and 3-*O*-β-d-glycosyl aesculin and PI were visualized with a 405-nm and a 488-nm laser, respectively.

Chemical changes after cellular uptake were determined by HPLC. RAW 264.7 cells (2 × 10^6^ cells/ml) were treated with 50 or 100 μM of 3-*O*-β-d-glycosyl aesculin as above and lysed by freezing and thawing using the liquid nitrogen three times. After centrifugation, the supernatant were filtered by 0.2 μm syringe filter and analyzed by HPLC.

### Animal cell culture

HEK 293 and RAW 264.7 cells (ATCC; Rockville, MD, USA) were cultured in Dulbecco’s Modified Eagle Medium supplemented with 10% (v/v) heat-inactivated FBS (Thermo, MA, USA), 100 U/ml penicillin, and 100 μg/ml streptomycin (Invitrogen; Carlsbad, CA, USA), at 37 °C under 5% CO_2_ in a humidified culture chamber. Nrf2 reporter cell lines were prepared as previously described^16^.

### Luciferase assay

Nrf2 reporter cells (5 × 10^5^ cells/well) under G418 (100 μg/ml) were incubated with various amounts of aesculin and 3-*O*-β-d-glycosyl aesculin for 16 h. Total cell lysate was prepared, and luciferase activity was measured by a luciferase assay kit (Promega; Madison, WI, USA) with the manufacturer’s instructions. Luciferase activity was normalized to the amount of total protein of the cell extract. The experiment was performed in triplicate.

### Western blotting

RAW 264.7 cells (1 × 10^6^ cells/well) were treated with sulforaphane, aesculin, or 3-*O*-β-d-glycosyl aesculin. Nuclear proteins were isolated by using an NE-PER nuclear extraction kit with the manufacturer’s protocol (Thermo Scientific; IL, USA). The amount of protein was determined by the Bradford assay (Bio-Rad). Equal amounts of protein were separated by 8% NuPAGE^®^ Bis-Tris gel electrophoresis (Invitrogen) in MOPS running buffer and transferred to polyvinyldifluoride (PVDF) membranes (Bio-Rad). Membranes were incubated with 1% bovine serum albumin and then with antibodies overnight at 4 °C (1:1000 dilution for Nrf2 or 1:5000 for HA). The membranes were stripped by a western blot stripping solution per the protocol by the manufacturer (Thermo Scientific) and reprobed with anti-lamin A/C antibody (1:5000 dilution). The proteins of interest were detected by a chemiluminescence substrate (SuperSignal West Femto; Thermo Scientific).

### Ubiquitination assay

Transfected with expression vectors of HA-Ub, V5-Nrf2, and FLAG-Keap1, HEK 293 cells were treated with 3-*O*-β-d-glycosyl aesculin for 8 h, along with 10 μM of MG132 (Sigma Aldrich) for 3 h prior to cell harvest. Cytosolic extracts were prepared, to which 1 μg of the antibody against HA or Nrf2 was added for immunoprecipitation. Immune complex was captured with protein A-sepharose (Invitrogen) and analyzed by western blotting for Nrf2 or ubiquitin with α-Nrf2 or α-HA antibody (1:5000 dilution). Equal loading was ensured by western blotting for β-actin.

### Semi-quantitative reverse transcription-polymerase chain reaction (RT-PCR) analysis

Total RNA was isolated with the QIAGEN RNeasy^®^ mini kit (Qiagen) according to the manufacturer’s instructions. 2 μg of RNA was reverse-transcribed by M-MLV reverse transcriptase (Promega) to generate cDNA. The quantity of each mRNA was determined by using end-point dilution PCR, including three serial 1 to 5 dilutions (1:1, 1:5, 1:25, and 1:125) of RT products prior to PCR amplification. To eliminate genomic DNA contamination, equal amounts of total RNA from each sample were PCR amplified without RT reaction. A portion of the cDNA was amplified by PCR with a set of specific primers as follows: the forward and the reverse primers for NQO-1 were 5′-GCAGTGCTTTCCATCACCAC-3′ and 5′-TGGAG TGTGCCCAATGCTAT-3′, respectively; the primers for HO-1 were 5′-TGAAGGAGGCCACCAAGGAGG-3′ and 5′-AGAGGTCACCCAGGTAGCGGG-3′, respectively; the primers for GCLC were 5′-CACTGCCAGAACACAGACCC-3′ and 5′-ATGGTCTGGCTGAGAAGCCT-3′, respectively; and the primers for GAPDH were 5′-GGAGCCAAAAGGGTCATCAT-3′ and 5′-GTGATGGCATGGACTGTGGT-3′, respectively. *Taq*PCRx DNA polymerase, Recombinant (Invitrogen) and the manufacturer’s protocol were used for PCR reaction. The reaction conditions were as follows: an initial denaturation at 95 °C for 5 min followed by 25 cycles of denaturation for 30 sec at 95 °C, annealing for 30 sec at 55 °C and extension for 40 sec at 72 °C with a final extension for 7 min at 72 °C. Amplicons were separated in 1.5% agarose gels in 1× TBE buffer at 100 V for 30 min, stained with GRgreen (Biolabo, châtel-St-Denis, Switzerland) and visualized under LED light. Glyceraldehyde-3-phosphate dehydrogenase (GAPDH) was used as internal controls to evaluate relative expressions of NQO-1, GCLC, and HO-1. The expression of each gene relative to GAPDH expression was determined by using the densitometric analysis software Image J (NIH; Bethesda, MD, USA).

### Measurement of intracellular reactive oxygen species (ROS)

Production of intracellular ROS in RAW 264.7 cells was measured by 5-(and-6)-carboxy-2′,7′ dichlorodihydro-fluorescein diacetate (carboxy-H_2_DCFDA; Molecular Probes; Eugene, OR, USA). Briefly, after various treatments, RAW 264.7 cells (1 × 10^6^ cells/well) were further treated with 100 μM carboxy-H_2_DCFDA in a cell culture medium and incubated at 37 °C for 30 min. After incubation, the cells were washed with PBS, and then fluorescence was measured using the BD FACS Canto II system (BD Biosciences; San Jose, CA, USA) at the excitation wavelength of 488 nm and the emission wavelength of 525 nm. At least 100,000 live events were collected for analysis. Data files were analyzed using FlowJo software (Tree Star, San Carlos, CA, USA).

### ALI mouse model and survival study

Wild type C57BL/6 (Jackson laboratory, Bar Harbor, ME, USA) and Nrf2 knockout (KO) mice (C57BL/6 background)^13^ were inbred in a specific pathogen-free (SPF) facility at Pusan National University, Yangsan, Korea. Animals were housed in certified, standard laboratory cages, and fed with food and water *ad libitum* prior to the experiment. Male mice aged between 7 to 10 weeks old were used for the study.

All experimental procedures followed the NIH of Korea Guidelines for the Care and Use of Laboratory Animals, and all experiments were approved by the Institutional Animal Care and Use Committee of Pusan National University (protocol number: PNU-2010-00028). After anesthetizing with Zoletil (Virbac, Carros cedex, France), mice received a single dose of 10 mg LPS/kg body weight for the induction of ALI. 3-*O*-β-d-glycosyl aesculin (0.15, 1.5, and 15 μg/kg) in PBS was loaded in a micro-sprayer (Model IA-1C, Penn-Century Inc., USA) and delivered in aerosol to the lung via intratracheal (i.t.) passage 2 h after the LPS administration. Bronchoalveolar lavage (BAL) and total and differential cell counting in BAL fluid were performed 24 h after LPS treatment, as described elsewhere^30^. Three hundred cells in total were counted, and one hundred of the cells in each microscopic field were scored. The mean number of cells per field was reported. For collecting lung tissue, mice were perfused with saline and the whole lung was inflated with fixatives. After paraffin embedding, 5 μm sections were cut and stained with a hematoxylin and eosin (HE) staining method. Three separate HE-stained sections were evaluated in 200X microscopic magnifications per mouse.

For survival studies, mice (n = 20 per group) received a lethal dose of LPS: i.p injection of a mixture of LPS (30 mg/kg body weight) and d-(+)-galactosamine hydrochloride (500 mg/kg body weight; Sigma). At 2 h after the injection, the mice received i.t. spraying of 1.5 μg/kg of 3-*O*-β-d-glycosyl aesculin. Survival of the mice was monitored for up to 4.5 days.

### Statistical analysis

Statistical analysis of results (*n* = 3 independent experiments) was performed using PASW Statistics Data Editor v18 Korean, SPSS Inc, Chicago, IL, USA. Values are expressed as the mean ± SEM. Student’s t-test and one-way analysis of variance (ANOVA) tests with Tukey’s post hoc test were applied for comparison of the means, and Kaplan-Meier estimate with log-rank test was used for survival analysis. *P* values less than 0.05 were considered statistically significant.

## Additional Information

**How to cite this article**: Kim, K. H. *et al*. Glycosylation enables aesculin to activate Nrf2. *Sci. Rep.* **6**, 29956; doi: 10.1038/srep29956 (2016).

## Supplementary Material

Supplementary Information

## Figures and Tables

**Table 1:** ^1^H and ^13^C NMR analyses of aesculin transglycosylation product.

	Aesculin		3-*O*-β-d-glycosyl aesculin	
	H	C	H	C
H, C-aromatic				
1	—	160.7	—	162.3
2	6.25	111.5	6.11	108.0
3	7.87	144.3	7.87	146.4
4	—	110.8	—	106.8
5	—	148.5	—	147.0
6	7.42	102.6	7.20	102.9
7	—	150.3	—	152.8
8	—	142.8	—	146.4
9	6.85	112.4	6.62	113.3
H, C-glucose				
1′	5.03	101.8	5.04	101.6
2′	3.56	74.1	3.57	75.7
3′	3.76	77.2	3.76	84.0
4′	3.86	70.6	3.87	69.8
5′	3.48	76.8	3.48	76.2
6′	3.80	61.8	3.82	60.9
	3.95		3.96	
1″			5.06 (d, *J* = 7.6 Hz)	104.9
2″			3.53	73.7
3″			3.66	76.2
4″			3.42	72.9
5″			3.68	76.0
6″			3.77	60.8
			3.85	
