# Supplementary material for: Glycosylation enables aesculin to activate Nrf2
Source: Sci Rep. 2016 Jul 15;6:29956. doi: 10.1038/srep29956 (PMC4945939; doi:10.1038/srep29956)
Supplement: Supplementary Information [file srep29956-s1.pdf]

Supplementary information

Glycosylation enables aesculin to activate Nrf2

Kyun Ha Kim[1], Hyunsu Park[2], Hee Jin Park[2], Kyoung-Hwa Choi[2], Ruxana T. Sadikot[3], Jaeho Cha[2,*], and Myungsoo Joo[1,*]

[1]School of Korean Medicine, Pusan National University, Yangsan 626-870, Korea
[2]Department of Microbiology, Pusan National University, Busan 609-735, Korea
[3]Section of Pulmonary and Critical Care Medicine, Atlanta Veterans Affairs Medical Center, Emory University, Decatur, GA30033, U.S.A.

[*]E-mail address: mjoo@pusan.ac.kr

Contents

Supplementary figures 1-3

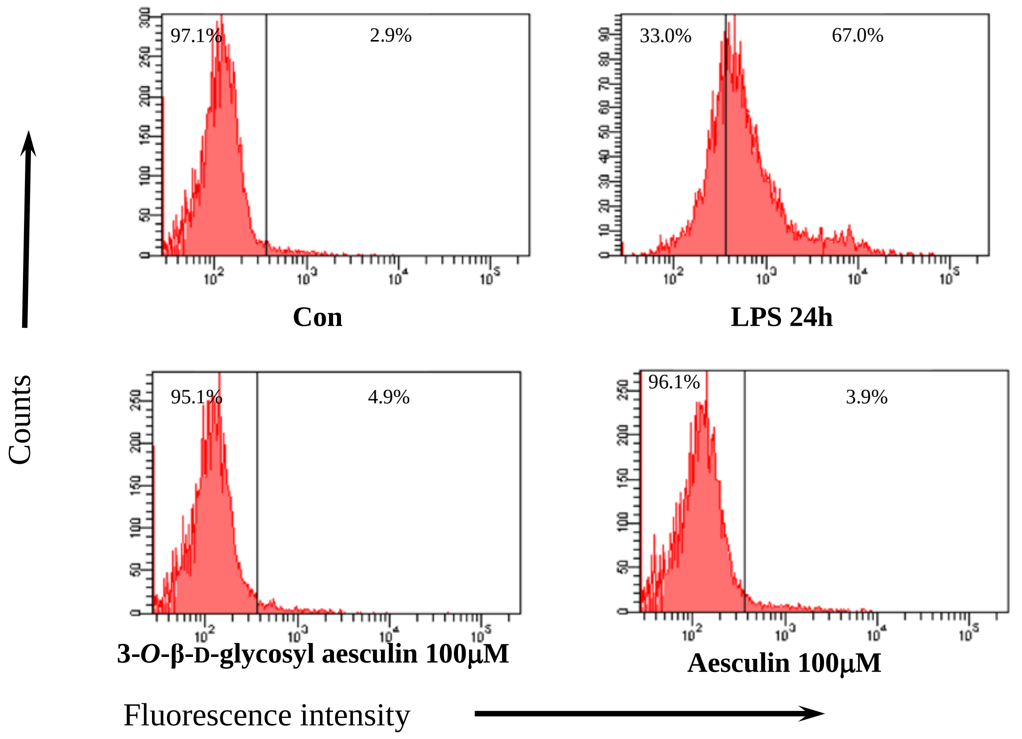

Supplementary figure S1.

Intracellular ROS production by 3-*O*-β-D-glycosyl aesculin and aesculin.

RAW 264.7 cells were treated with 100 μM of 3-*O*-β-D-glycosyl aesculin or aesculin for 16h, and intracellular ROS in the RAW 264.7 cells were analyzed by FACS. As a positive control for ROS, RAW 264.7 cells were similarly treated with LPS (1μg/ml). The percentage of cells producing intracellular ROS is shown on the right panel.

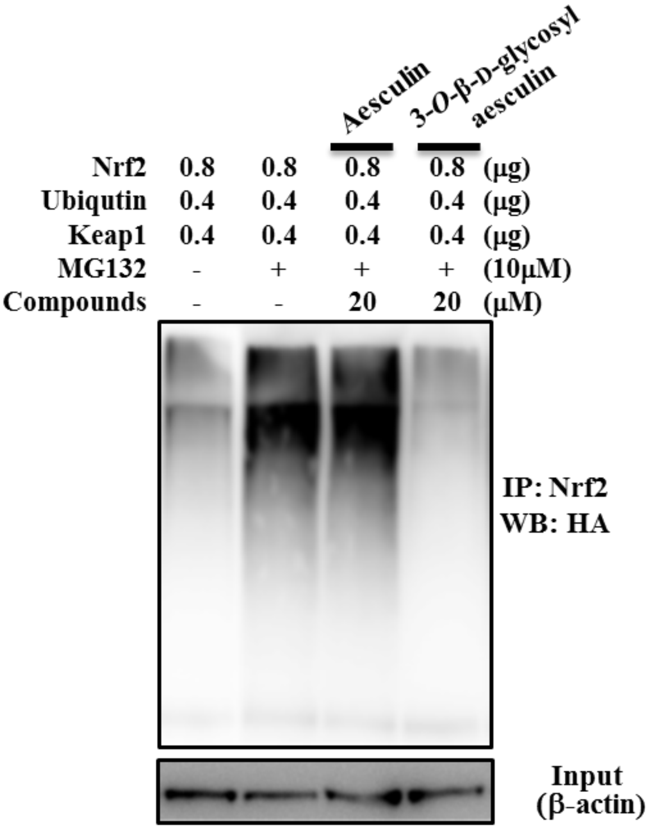

Supplementary figure S2.

3-*O*-β-D-glycosyl aesculin, but not aesculin, blocks ubiquitination of Nrf2.

HEK 293 cells, transfected with plasmids encoding Nrf2, HA-ubiquitin, and Keap1, were treated with aesculin or 3-*O*-β-D-glycosyl aesculin. Immunoprecipitation of Nrf2 and subsequent western blotting for HA (ubiquitin) were performed to reveal the ubiquitinated Nrf2.

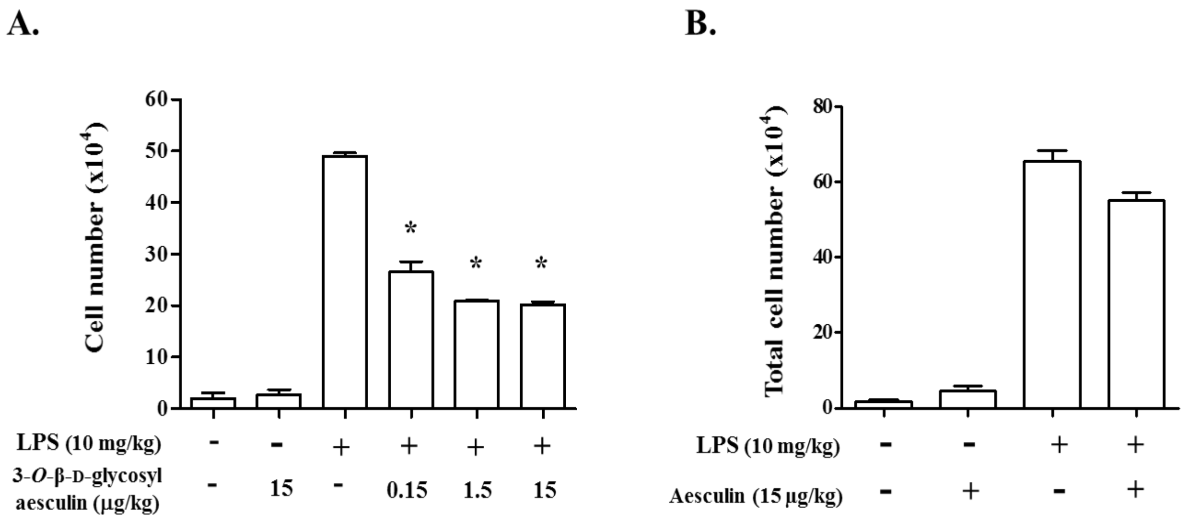

Supplementary figure S3.

3-*O*-β-D-glycosyl aesculin, but not aesculin, suppresses lung inflammation in ALI mice. (A) C57BL/6 mice (n = 5 per group) received an i.p. LPS (3rd to 6th lanes) and 2 h later 0.15 μg/kg, 1.5 μg/kg, or 15 μg/kg of i.t. 3-*O*-β-D-glycosyl aesculin, respectively (4th to 6th lanes). BAL was performed for counting total cells infiltrated to mouse lungs. (B) In similar experiments, mice (n = 5 per group) received an i.p. LPS (3rd and 4th lanes) and 2 h later 15 μg/kg of i.t. aesculin (2nd and 4th lanes). BAL was performed for counting total cells infiltrated to mouse lungs. Data represent the mean ± SEM of three independent counting. *P was < 0.05, compared with mice treated with LPS only.
